# Association Between Tonsillectomy and Outcomes in Patients With Immunoglobulin A Nephropathy

**DOI:** 10.1001/jamanetworkopen.2019.4772

**Published:** 2019-05-31

**Authors:** Keita Hirano, Keiichi Matsuzaki, Takashi Yasuda, Masako Nishikawa, Yoshinari Yasuda, Kentaro Koike, Shoichi Maruyama, Takashi Yokoo, Seiichi Matsuo, Tetsuya Kawamura, Yusuke Suzuki

**Affiliations:** 1 Division of Nephrology, Department of Internal Medicine, Ashikaga Red Cross Hospital, Ashikaga, Japan; 2 Division of Nephrology and Hypertension, Department of Internal Medicine, Jikei University School of Medicine, Tokyo, Japan; 3 Department of Nephrology, Juntendo University Faculty of Medicine, Tokyo, Japan; 4 Kyoto University Health Service, Kyoto, Japan; 5 Department of Internal Medicine, Kichijoji Asahi Hospital, Kyoto, Japan; 6 Clinical Research Support Center, Jikei University School of Medicine, Tokyo, Japan; 7 Department of Nephrology, Nagoya University, Nagoya, Japan

## Abstract

**Importance:**

Immunoglobulin A nephropathy is a major cause of end-stage renal disease worldwide; previous methods of medical management, including use of renin-angiotensin system inhibitors and corticosteroids, remain unproven in clinical trials.

**Objective:**

To investigate the possible association between tonsillectomy and outcomes in patients with IgA nephropathy.

**Design, Setting, and Participants:**

This cohort study included 1065 patients with IgA nephropathy enrolled between 2002 and 2004 and divided into 2 groups, those who underwent tonsillectomy and those who did not. Initial treatments (renin-angiotensin system inhibitors or corticosteroids) within 1 year after renal biopsy were also evaluated. A 1:1 propensity score matching was performed to account for between-group differences and 153 matched pairs were obtained. Follow-up concluded January 31, 2014. Analysis was conducted between September 11, 2017, and July 31, 2018.

**Exposure:**

Tonsillectomy.

**Main Outcomes and Measures:**

The primary outcome was the first occurrence of a 1.5-fold increase in serum creatinine level from baseline or dialysis initiation. Secondary outcomes included additional therapy with renin-angiotensin system inhibitors or corticosteroids initiated 1 year after renal biopsy and adverse events.

**Results:**

In 1065 patients (49.8% women; median [interquartile range] age, 35 [25-52] years), the mean (SD) estimated glomerular filtration rate was 76.6 (28.9) mL/min/1.73 m^2^ and the median (interquartile range) proteinuria was 0.68 (0.29-1.30) g per day. In all, 252 patients (23.7%) underwent tonsillectomy within 1 year after renal biopsy and 813 patients (76.3%) did not undergo tonsillectomy. The primary outcome was reached by 129 patients (12.1%) during a median (interquartile range) follow-up of 5.8 (1.9-8.5) years. In matching analysis, tonsillectomy was associated with primary outcome reduction (hazard ratio, 0.34; 95% CI, 0.13-0.77; *P* = .009). In subgroup analyses, benefit associated with tonsillectomy was not modified by baseline characteristic differences. Those undergoing tonsillectomy required fewer additional therapies 1 year following renal biopsy (adjusted hazard ratio, 0.37; 95% CI, 0.20-0.63; *P* < .001) without increased risks for adverse events, except transient tonsillectomy-related complications.

**Conclusions and Relevance:**

This study found that tonsillectomy was associated with a lower risk of renal outcomes in patients with IgA nephropathy. The potential role of tonsillectomy should be considered for preventing end-stage renal disease in patients with IgA nephropathy.

## Key Points

**Question:**

Is there an association between undergoing tonsillectomy within 1 year of the initial diagnosis of IgA nephropathy and the subsequent renal outcome?

**Findings:**

In this cohort study of 1065 Japanese patients with IgA nephropathy diagnosed between 2002 and 2004, the matched patients who underwent tonsillectomy had a lower risk of renal events than those who did not undergo the procedure.

**Meaning:**

Tonsillectomy may improve renal survival rates in patients with IgA nephropathy independent of conventional therapy using renin-angiotensin system inhibitors and corticosteroids.

## Teaser

This cohort study investigates the association between tonsillectomy and renal outcomes in patients with IgA nephropathy in Japan.

## Introduction

Immunoglobulin A nephropathy (IgAN) is the most prevalent primary chronic glomerulonephritis worldwide, causing end-stage renal disease in up to 40% of affected patients.^1^ In patients who reach end-stage renal disease, the mortality rate increases 5-fold, with cardiovascular events accounting for 45% of all deaths.^2^ The genetic risk for IgAN increases with the eastward distance from Africa.^3^ The relative IgAN frequency among all primary glomerulonephritis cases in biopsy databases tends to be highly variable, ranging from 50% in China and Japan^4,5^ to 10% to 35% across Europe^6,7^ and decreasing to 5% in the Middle East.^8^ As partially described in the Kidney Disease: Improving Global Outcomes (KIDGO) clinical practice guideline, studies from Asia^9^ and Europe^10^ have reported the utility of corticosteroids and renin-angiotensin system inhibitors (RASi) to treat IgAN. However, the risk-to-benefit ratio for corticosteroids in IgAN has been challenged,^11,12^ and alternative therapeutic options are needed.

The major IgAN manifestations are renal IgA deposition and mesangial proliferation.^13^ Impaired immune regulation, characterized by the overproduction of aberrantly glycosylated and polymeric IgA 1, plays an important role in IgAN pathogenesis.^14,15^ The most evident connection between IgAN and the mucosa–bone marrow pathway is visible hematuria with acute upper respiratory tract infections.^16^ Site-specific steroids have been proposed to improve renal prognoses.^17^ A novel targeted-release formulation of the corticosteroid budesonide, designed to be delivered to the distal ileum, exhibited an antiproteinuric effect and preserved patients’ renal function in a phase 2b randomized clinical trial. Therefore, site-specific therapeutic strategies targeting the mucosa–bone marrow–kidney axis in patients with IgAN may prove effective against IgAN.

Two randomized clinical studies on Asian patients (from Japan in 2014^18^ and China in 2016^19^) independently demonstrated that tonsillectomy with corticosteroid treatment improves proteinuria. However, the association between tonsillectomy and long-term renal prognosis remains controversial. In 2001, Hotta et al^20^ reported that tonsillectomy was an independent predictor of the lack of renal damage progression in a study including 329 Japanese patients with IgAN. Chen et al^21^ reported a favorable but nonsignificant effect of tonsillectomy on long-term renal prognosis in Chinese patients with IgAN. Furthermore, tonsillectomy is currently performed in adult patients with IgAN in more than 50% of institutions in Japan.^22^ Conversely, the European Validation Study of the Oxford Classification of IgAN (VALIGA), a multicenter retrospective study including 1147 patients with IgAN in Europe, reported no significant effect of tonsillectomy on long-term prognosis.^23^ However, the predictive power for tonsillectomy in the VALIGA study was limited to only 17 patients (1.4%) who received tonsillectomy after renal biopsy during the follow-up.^23^ Genetic studies may address the rationale for tonsillectomy in IgAN, and a genome-wide association study^24^ from Danish health registers demonstrated the association of 1 genetic variant with tonsillectomy risk (due to severe tonsillitis or massive tonsillar hypertrophy) and IgAN susceptibility. Interestingly, the same variant was also associated with IgAN progression in other studies.^3,25^

We conducted a multicenter study, recruiting participants from across Japan (Japanese Nationwide Retrospective Cohort Study in IgAN [JNR-IgAN]), a country with the highest IgAN prevalence and a long history of performing tonsillectomy as part of its treatment.^4^ Owing to the retrospective nature of the study, we used Kaplan-Meier analyses and Cox regression models after propensity matching to explore the association between tonsillectomy and the primary outcome of a 1.5-fold increase in serum creatinine from baseline or dialysis initiation.

## Methods

As part of the research study of the Progressive Kidney Disease Study Group funded by the Japanese Ministry of Health, Labour, and Welfare in 2012, the St Marianna University School of Medicine Institutional Review Board on Human Research, acting as the main committee for this study, together with each participating institute’s local ethics committee, approved the project. We used an anonymized data set for research purposes as per protocol. Hence, the need for written informed consent from participants was waived. The study followed the Strengthening the Reporting of Observational Studies in Epidemiology (STROBE) reporting guideline for cohort studies.

### Study Population

The data for this nationwide retrospective study were obtained from the JNR-IgAN cohort. All patients with IgAN were eligible to be included in the JNR-IgAN cohort if they were older than 18 years and had received the IgAN diagnosis by an initial renal biopsy between 2002 and 2004 at 1 of 42 universities or leading community hospitals located in major cities across Japan (eTable 1 in the Supplement). Among the 1174 patients with IgAN in the JNR-IgAN cohort, we excluded data from patients receiving tonsillectomy prior to renal biopsy and those with missing data (such as baseline serum creatinine, treatment after diagnosis, and outcomes). We used anonymized patient records to prevent personal information identification. The final follow-up date was January 31, 2014. The dates of the analysis were September 11, 2017, to July 31, 2018.

### Exposures

Based on the currently available therapy options at the time of diagnosis, we categorized the initial treatments (initiated within 1 year after renal biopsy) as those including tonsillectomy (T1), those without tonsillectomy (T0), and those including corticosteroids (S1 for regimens without pulses, S2 for regimens including pulses, and S0 for those without corticosteroid therapy).^20,26^ Furthermore, if the patients had already received RASi at the time of renal biopsy or initiated the treatment within 1 year after renal biopsy, we defined their treatment as including baseline RASi. The exposure of interest in our study was tonsillectomy (T1 vs T0).

### Outcomes

The primary outcome was a renal event, defined as the first occurrence of a 1.5-fold increase in serum creatinine from baseline or dialysis initiation.^27^ Secondary outcomes were additional therapy with RASi or corticosteroid initiated 1 year after the renal biopsy and adverse event occurrences (eg, death, infectious disease, tonsillectomy complications).

### Important Covariates

We included the following covariates in baseline characteristics (Table 1), in which baseline was at the time of renal biopsy: age, sex, body mass index, diabetes presence or absence, mean arterial pressure (MAP), estimated glomerular filtration rate (eGFR), proteinuria, occult blood in urine, uric acid, total cholesterol, IgA, and complement 3 (C3). The following equation was used to estimate MAP: MAP (mmHg) = 1/3 × pulse pressure + diastolic blood pressure. The following equation (modified from the Modification of Diet in Renal Disease equation^28^) was used to estimate eGFR based on serum creatinine value: eGFR (mL/min/1.73 m^2^) = 194 × (serum creatinine [mg/dL])^−1.094^ × (age [years])^−0.287^ ( × 0.739 if female).

**Table 1.** Baseline Characteristics and Follow-up Data From Total Cohort Population and Groups According to Tonsillectomy Category

Characteristic	Overall (N = 1065)	Tonsillectomy		*P* Value
		T1 (n = 252)	T0 (n = 813)	
Baseline^a^				
Age, median (IQR), y	35 (25-52)	29 (23-41)	39 (27-54)	<.001
Women, No. (%)	530 (49.8)	146 (57.9)	384 (47.2)	.003
Diabetes, No. (%)	49 (4.6)	6 (2.4)	43 (5.3)	.05
Body mass index, mean (SD)^b^	22.5 (3.5)	22.1 (3.6)	22.7 (3.4)	.04
Arterial pressure, mean (SD), mm Hg	92.3 (13.2)	89.2 (13.1)	93.2 (13.1)	<.001
Estimated glomerular filtration rate, mean (SD), mL/min/1.73 m^2^	76.6 (28.9)	83.7 (29.1)	74.5 (28.5)	<.001
Proteinuria, median (IQR), g/d	0.68 (0.29-1.30)	0.71 (0.33-1.30)	0.65 (0.28-1.30)	.67
Urine occult ≥3, No. (%)	577 (54.2)	153 (60.7)	424 (52.2)	.02
Uric acid, mean (SD), mg/dL	5.9 (1.5)	5.7 (1.6)	5.9 (1.5)	.12
Initial treatments, No. (%)				
Renin-angiotensin aldosterone system inhibitors	595 (55.9)	106 (42.1)	489 (60.1)	<.001
Corticosteroid therapy				<.001
No steroid therapy	574 (53.9)	54 (21.4)	520 (64.09)	
Oral steroid therapy without pulse regimen	204 (19.2)	32 (12.7)	172 (21.1)	
Oral steroid therapy with pulse regimen	287 (26.9)	166 (65.9)	121 (14.9)	
Follow-up				
Time, median (IQR), y	5.8 (1.9-8.5)	5.3 (1.7-8.2)	6.0 (2.0-8.6)	.18
Patients who reached primary outcomes, No. (%)	129 (12.1)	11 (4.4)	118 (14.5)	<.001
Additional therapy, No. (%)				<.001
Renin-angiotensin aldosterone system inhibitors	82 (7.7)	15 (6.0)	67 (8.2 )	
Steroid therapy	56 (5.3)	3 (1.2)	53 (6.5)	
Renin-angiotensin aldosterone system inhibitors and steroid therapy	15 (1.4)	0	15 (1.9)	
Adverse events, No. (%)				
Death	6 (0.6)	0	6 (0.7)	.35
Infectious disease	19 (1.8)	1 (0.4)	18 (2.2)	.06
Diabetes	12 (1.1)	1 (0.4)	11 (1.4)	.31
Malignant neoplasm	8 (0.8)	0	8 (1.0)	.21
Cardiovascular disease	6 (0.6)	0	6 (0.7)	.35
Cerebral infarction	1 (0.1)	0	1 (0.1)	.58
Peptic ulcer	7 (0.7)	1 (0.4)	6 (0.7)	.56
Psychogenic disorder	8 (0.8)	0	8 (1.0)	.21
Cataract	7 (0.7)	0	7 (0.9)	.21
Tonsillectomy-related events	7 (0.7)	7 (2.8)	0	<.001
Other event	15 (1.4)	1 (0.4)	14 (1.7)	.12

Abbreviations: IQR, interquartile range; T1, patients who underwent tonsillectomy; T0, patients who did not undergo tonsillectomy.

SI conversion factor: To convert uric acid to μmol/L, multiply by 59.485.

^a^ Baseline was at the time of renal biopsy.

^b^ Calculated as weight in kilograms divided by height in meters squared.

### Statistical Analysis

To summarize baseline characteristics, we used means with standard deviations or medians with interquartile ranges (IQRs) for continuous variables and percentages for categorical variables. We compared normally distributed, nonparametric, and categorical variables using the 1-way analysis of variance, the Wilcoxon test, and the χ^2^ test, respectively, except for standardized differences. To explore the association between tonsillectomy and the primary outcome, we used Kaplan-Meier analysis and Cox regression models after propensity score matching. We used 2 methods for matching propensity scores: 1:1 matching and inverse probability of treatment weighted estimators for average treatment effect on the treated.^29^ A multiple logistic regression model assigned propensity scores using the covariates listed in eTable 2 in the Supplement. For the 1:1 matching step, we chose the nearest neighbor matching with a caliper width of 1/4 logits of the standard deviation. After matching or weighting by the propensity score, we assessed the balance of each covariate by the absolute standardized difference and paired test, and we considered any standardized difference greater than 10% or *P* value greater than .05 as indicating a meaningful imbalance. To evaluate the interaction between tonsillectomy and each covariate in relation to the outcome, we used stratified Cox regression models to estimate hazard ratios (HRs) in different groups. Specifically, we conducted an exploratory analysis in subgroups based on patient characteristics, including demographics, eGFR, proteinuria, hematuria, and RASi. To better understand the association between tonsillectomy and corticosteroid therapy regarding the outcome, we performed a different stratified analysis, as the corticosteroid therapy included different regimens. First, we categorized the entire cohort into 6 groups based on the initial treatment with tonsillectomy (T1 or T0) and corticosteroids (S2, S1, or S0) resulting in T1S2, T1S1, T1S0, T0S2, T0S1, and T0S0 groups. Second, we estimated combined HRs (T1 vs T0) in relation to the outcomes in various subgroups by comparing the primary HRs in those 6 categories with the T0S0 reference group; namely, S0 group without corticosteroid therapy (T1S0 and T0S0), S1 with oral corticosteroid without pulse therapy (T1S1 and T0S1), S2 with oral corticosteroid and pulse therapy (T1S2 and T0S2), and S1 and S2 with any corticosteroid therapy (T1S1, T0S1, T1S2, and T0S2). Third, we calculated the differences between each combined HR in S1, S2, or S1 and S2, and that in S0. During these sequential steps, we used the CLASS, MODEL, CONTRAST, and HAZARDRATIO options in the SAS statistical software version 9.2 (SAS Institute Inc) PHREG procedure. We estimated that enrolling 107 patients would provide 80% power to detect a difference in tonsillectomy category with a 2-sided significance level of .05 in the analysis of the primary outcome, assuming an expected result of a 0.33 HR.^30^ For all models, we graphically verified the proportionality of hazards for the Cox proportional hazards assumption using log plots. For missing values, we used the multivariate imputation method (eTable 3 in the Supplement). We performed all statistical analyses using the JMP statistical software version 13.2.0 (SAS Institute Inc) and SAS version 9.2.

## Results

### Baseline Characteristics and Outcomes

In 1065 patients (49.8% women; median [interquartile range] age, 35 [25-52] years), the mean (SD) estimated glomerular filtration rate was 76.6 (28.9) mL/min/1.73 m^2^ and the median (interquartile range) proteinuria was 0.68 (0.29-1.30) g per day. In all, 252 patients (23.7%) underwent tonsillectomy within 1 year after renal biopsy and 813 patients (76.3%) did not undergo tonsillectomy. Prior to our study, 109 individuals in the JNR-IgAN cohort (9.3%) had been excluded for the following reasons: not fulfilling the biopsy date inclusion criterion (7 participants), tonsillectomy prior to the renal biopsy (23 participants), data missing on baseline serum creatinine (1 participant), and data missing on final serum creatinine or outcomes (78 participants). Finally, we included data from 1065 patients. Table 1 shows their baseline characteristics and follow-up data (eTable 3 and eFigure 1 in the Supplement). In contrast to the largest European IgAN cohort (VALIGA cohort), this Japanese cohort had lower proteinuria (0.68 vs 1.30 g per day) and lower MAP (92.3 vs 98.0 mm Hg) at diagnosis, suggesting that diagnoses are made during earlier stages in Japan. Another important difference between the 2 cohorts was RASi use, which was unrestricted in the VALIGA cohort (91.5% patients used them following biopsy) but reserved for patients with hypertension in our cohort (55.9% of patients). Despite the relatively mild disease in the Japanese cohort, we found clear evidence of glomerular inflammation, with more than half of the patients presenting the highest-grade hematuria possible.

During the median (IQR) follow-up of 5.8 (1.9-8.5) years, 129 patients (12.1%) among the overall population reached the primary outcome, defined as a 1.5-fold increase in serum creatinine level from baseline or dialysis initiation. A further 153 patients (14.4%) received additional therapy 1 year after the renal biopsy with RASi and/or corticosteroid therapy.

### Favorable Association Between Tonsillectomy and Outcome

**Figure 1.**
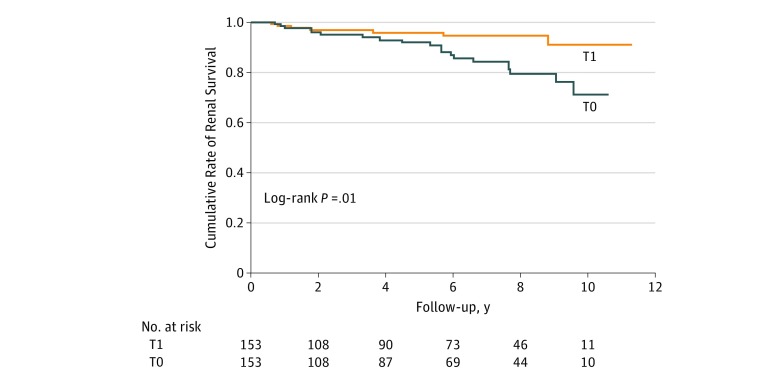
Comparison of the Cumulative Rates of Renal Survival Between the T1 and T0 Groups After Propensity Score Matching T1 indicates patients who underwent tonsillectomy; T0, patients who did not undergo tonsillectomy.

**Figure 2.**
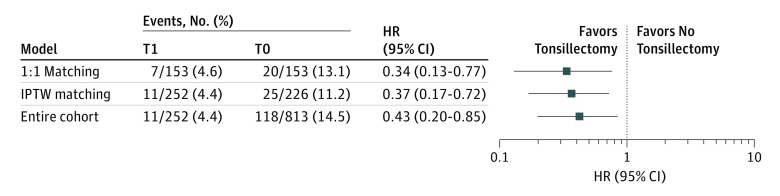
Association Between Tonsillectomy and Primary Outcome in Various Multivariate Models Hazard ratios (HRs) and 95% confidence intervals for primary events consistently and significantly showed that tonsillectomy was associated with a favorable renal outcome in various multivariate models, including a 1:1 propensity matching model, the inverse probability of treatment–weighted (IPTW) matching model, and a simple multivariate model of the entire cohort. The HRs and 95% CIs (tonsillectomy vs no tonsillectomy) are presented as a log-log plot. The matching model was created by a propensity score using a caliper (*a* = 0.30 and *c* = 0.51) (Table 2; eTable 2 in the Supplement). The IPTW model was the mean treatment effect on those treated (eTable 4 in the Supplement). We adjusted the standard Cox proportional hazards model according to age, sex, body mass index, diabetes presence or absence, mean arterial pressure, estimated glomerular filtration rate, proteinuria, urine occult, uric acid, total cholesterol, IgA, complement 3, renin-angiotensin system inhibitor use, and corticosteroid therapy. Supplementary predictive values of these factors are shown in eTable 5 in the Supplement. T1 indicates patients who underwent tonsillectomy; T0, patients who did not undergo tonsillectomy.

**Figure 3.**
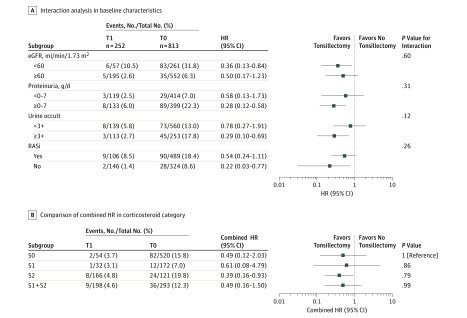
Association Between Tonsillectomy and Primary Outcome in Subgroups for the Baseline Characteristics and Initial Treatments A, No interaction between baseline clinical parameters and tonsillectomy was found in the overall cohort. Stratified analyses suggest that almost all clinical subgroups tended to have an event-free survival benefiting from tonsillectomy, independently of their estimated glomerular filtration rate (eGFR), proteinuria, hematuria extent, or renin-angiotensin aldosterone system inhibitors (RASi) use. B, Despite the wide 95% CI, the combined tonsillectomy hazard ratios (HRs) consistently exhibited an association with favorable outcomes across various corticosteroid subgroups. Each combined HR in oral steroid therapy without pulse regimen (S1), oral steroid therapy with pulse regimen (S2), or S1 and S2 did not significantly differ from that in no steroid therapy (S0). T1 indicates patients who underwent tonsillectomy; T0, patients who did not undergo tonsillectomy.

Our analysis demonstrated the association between tonsillectomy and a lower risk of the primary outcome. Owing to significant differences between baseline characteristics in patients with T1 and T0, we conducted propensity score matching before evaluating our primary outcome. Thus, within the overall cohort, we were able to pair 153 patients in T1 to 153 patients in T0 (Table 2). All differences between patients’ characteristics in T1 and those in T0 (Table 1) were accounted for once patients were matched to controls according to age, sex, diabetes presence or absence, body mass index, MAP, eGFR at renal biopsy, hematuria extent, RASi use, and corticosteroid therapy (Table 2). Our Kaplan-Meier survival curve and Cox regression model revealed significantly better renal survival in patients in the T1 group than in the T0 group (HR, 0.34; 95% CI, 0.13-0.77; *P* = .009) (Figure 1 and Figure 2). This independent association between tonsillectomy and a lower risk of the outcome was consistently confirmed in our inverse probability of treatment weighted model and in the entire cohort, using 2 increased sampling sizes from the entire cohort (Figure 2; eTable 4 and eTable 5 in the Supplement). All adjusted HRs on the standard multivariate regression model (at the bottom of Figure 2) are listed in eTable 5 in the Supplement. We found no interaction effects between baseline characteristics, including eGFR, proteinuria, hematuria extent, and RASi use (Figure 3A). Furthermore, the favorable association between tonsillectomy and outcome was not modified by the difference in the corticosteroid therapeutic categories S1, S2, S1 and S2, and S0 (Figure 3B; eTable 6 and eFigure 2 in the Supplement).

**Table 2.** Comparison of Baseline Characteristics Between the T1 and T0 Groups After Propensity Matching^a^

Baseline Characteristic^b^	T1 (n = 153)	T0 (n = 153)	*P* Value	Standardized Difference, %
Age, median (IQR), y	31 (24-46)	30 (23-44)	.64	5.3
Women, No. (%)	87 (56.9)	83 (54.3)	.65	5.3
Diabetes, No. (%)	5 (3.3)	5 (3.3)	>.99	0
Body mass index, mean (SD)	22.1 (3.5)	22.0 (3.9)	.87	1.9
Arterial pressure, mean (SD), mm Hg	90.0 (13.2)	89.3 (12.5)	.58	5.4
Estimated glomerular filtration rate at renal biopsy, mean (SD), mL/min/1.73 m^2^	79.4 (27.8)	80.3 (31.4)	.79	3
Proteinuria, median (IQR), g/d	0.79 (0.30-1.41)	0.62 (0.26-1.36)	.78	3.6
Urine occult ≥3, No. (%)	91 (59.5)	93 (60.8)	.82	2.7
Uric acid, mean (SD), mg/dL	5.7 (1.6)	5.7 (1.5)	.89	1.6
Initial treatments, No. (%)				
Renin-angiotensin aldosterone system inhibitors	73 (47.7)	74 (48.4)	.91	1.4
No steroid therapy	54 (35.3)	56 (36.6)	.81	2.7
Oral steroid therapy without pulse regimen	30 (19.6)	28 (18.3)	.77	3.3
Oral steroid therapy with pulse regimen	69 (45.1)	69 (45.1)	>.99	0

Abbreviations: IQR, interquartile range; T0, patients who did not undergo tonsillectomy; T1, patients who underwent tonsillectomy;

SI conversion factor: To convert uric acid to μmol/L, multiply by 59.485.

^a^ Caliper: *a* = 0.30, *c* = 0.51.

^b^ Baseline was at the time of renal biopsy, except for initial treatments, which was defined within 1 year after renal biopsy.

In addition to a favorable renal outcome, tonsillectomy was associated with fewer additional therapy requirements 1 year after the renal biopsy in the 1:1 matched cohort (HR, 0.36; 95% CI, 0.17-0.71; *P* = .003) and in the entire cohort (adjusted HR, 0.37; 95% CI, 0.20-0.63; *P* < .001).

### Adverse Events

Six patients in the T0 group died during the follow-up period, including 2 patients with lung cancer, 2 patients with gastric cancer, 1 patient with chronic obstructive pulmonary disease and pneumonia, and 1 patient with a ruptured abdominal aortic aneurysm (Table 1). Conversely, we observed no deaths in patients in the T1 group. In addition, we found 90 adverse events from 59 patients in the entire cohort, including infectious diseases, diabetes, malignant neoplasms, cardiovascular disease, cerebral infarction, peptic ulcer, psychogenic disorder, and transient tonsillectomy-related complications. Tonsillectomy did not increase the risk of adverse events except for transient tonsillectomy-related complications, which included postoperative hemorrhage or infection.

## Discussion

To our knowledge, this is the first nationwide multicenter cohort study evaluating the therapeutic value of tonsillectomy for the treatment of IgAN in Japanese patients. Our findings agree with those in a meta-analysis by Duan et al,^30^ which included 19 studies and a total of 3483 participants. Our 1:1 propensity matching model (Figure 1) found an HR of 0.34 (95% CI, 0.13-0.77), and the meta-analysis found a pooled odds ratio of 0.33 (95% CI, 0.16-0.69).^30^ Although the meta-analysis involved 2 studies from cohorts of European heritage, 17 studies were of Asian cohorts.^30^ It is important to differentiate the effect of tonsillectomy in the Japanese population and the effect seen in other populations, particularly in those of European descent, for whom the benefit of tonsillectomy may be lost. The meta-analysis by Duan et al^30^ found a point estimate odds ratio of 0.54 for tonsillectomy from the European VALIGA study, but the 95% CI of 0.14 to 2.04 was very wide, presumably owing to the low tonsillectomy rate in the VALIGA study (1.7%).^30^ In contrast, 23.7% of the patients in our Japanese cohort received tonsillectomy, and they displayed a wide range of baseline characteristics (age range, 18-68 years; eGFR range, 22.7-210.1 mL/min/1.73 m^2^; and proteinuria range, 0.01-4.22 g per day), reflecting the real-life clinical experience in Japan.

Galactose-deficient IgA and related immune complexes are reportedly involved in IgAN pathogenesis and progression. The immune cells responsible for galactose-deficient IgA production reside in the mucosal-associated lymphoid tissue, and the tonsils are a key component of this type of tissue.^14,15^ Analogous to the Peyer patches of the small intestines, the tonsils may offer an important therapeutic target in IgAN, and tonsillectomy may provide a therapeutic benefit independent of systemic or targeted corticosteroid therapy, as shown by our results (Figure 1, Figure 2, and Figure 3). Studies from Asia have already shown that tonsillectomy reduces the risk of recurring IgAN after induction of corticosteroid therapy in patients with IgAN and relapsed IgAN after renal transplantation.^19,31,32,33^ Our results may also partially support those studies because the patients who underwent tonsillectomy (T1) required fewer additional therapies 1 year after the renal biopsy than patients without tonsillectomy (T0). From another perspective, 595 patients (55.9%) received RASi in this study. The HR for tonsillectomy was 0.54 (95% CI, 0.24-1.11) in the RASi-treated group and 0.22 (95% CI, 0.03-0.77) in the untreated group (Figure 3A). These results confirm that tonsillectomy is associated with a favorable renal outcome independently from glomerular hypertension control provided by RASi.

The rate of complications following tonsillectomy in our study (2.8%), all of which were reversible with intervention, was comparable to those reported in the studies from Japan^34^ and the United States.^35^ Except for these reversible postoperative complications, tonsillectomy did not increase the risk of adverse events in our study; this further strengthens the idea that tonsillectomy may be used in clinical settings to reduce the risk of irreversible end-stage renal disease in patients with IgAN.

### Limitations

This study had limitations. First, as mentioned, the study cohort included patients who received additional treatment during the follow-up period. We evaluated the association between the initial treatment and the renal outcome for patients with treatments within the first year after renal biopsy. Therefore, the association between the primary outcome and additional treatments remains unknown. We did not include the covariates of urinalysis findings and renal function during the follow-up. Therefore, the exact effect of IgAN recurrence remains unknown. Results from the ongoing nationwide multicenter prospective cohort study (Japan IgA Nephropathy Cohort Study), which is collecting data from more than 1000 patients every 6 months during the follow-up period, are not yet available and will be reported separately. Second, the study cohort included only Japanese patients; therefore, the applicability of these findings to other populations is unknown. Racial differences in the susceptibility genes for IgAN have been suggested in genome-wide association studies.^3^ Third, the study cohort did not include pediatric patients aged younger than 18 years at diagnosis. Thus, the association between tonsillectomy for children diagnosed with IgAN and renal outcome is unclear. Fourth, we used the 1.5-fold increase in serum creatinine level from baseline values (the so-called soft end point) as the main and most relevant primary end point.^27^ Fifth, despite our comprehensive propensity risk scoring, this study may have been biased by unidentified confounding factors, although we made every attempt to account for those variables with the strongest support from the literature for influencing renal outcomes.

## Conclusions

This nationwide retrospective cohort study in Japan found that tonsillectomy is associated with improved renal survival rates in patients with IgAN. Further data from prospective studies, including the ongoing prospective Japan IgA Nephropathy Cohort Study, will provide additional evidence on longer-term outcomes following initial treatment with tonsillectomy in the coming years.

## Notes

Supplement. **eTable 1.** Participating Facilities **eTable 2.** Variables Used for Propensity Score Analysis **eTable 3.** Baseline Characteristics of the Study Cohort Before and After Imputation **eTable 4.** Comparison of Baseline Characteristics Between T0 and T1 Groups After IPTW **eTable 5. **A Simple Multivariate Model for the Primary Outcome in Entire Cohort **eTable 6.** Baseline Characteristics and Follow-up Data in Six Groups Categorized According to Tonsillectomy and Corticosteroid Therapy **eFigure 1.** Rate of Patients Defined by Tonsillectomy and Corticosteroid Therapy According to Renin Angiotensin System Inhibitors **eFigure 2.** Adjusted Hazard Ratios for the Primary Outcome According to Six Groups Categorized by the Combination of Tonsillectomy and Type of Corticosteroid Therapy
