# Supplementary material for: Association Between Tonsillectomy and Outcomes in Patients With Immunoglobulin A Nephropathy
Source: JAMA Netw Open. 2019 May 31;2(5):e194772. doi: 10.1001/jamanetworkopen.2019.4772 (PMC6547111; doi:10.1001/jamanetworkopen.2019.4772)
Supplement: Supplement. — eTable 1. Participating Facilities eTable 2. Variables Used for Propensity Score Analysis eTable 3. Baseline Characteristics of the Study Cohort Before and After Imputation eTable 4. Comparison of Baseline Characteristics Between T0 and T1 Groups After IPTW eTable 5. A Simple Multivariate Model for the Primary Outcome in Entire Cohort eTable 6. Baseline Characteristics and Follow-up Data in Six Groups Categorized According to Tonsillectomy and Corticosteroid Therapy eFigure 1. Rate of Patients Defined by Tonsillectomy and Corticosteroid Therapy According to Renin Angiotensin System Inhibitors eFigure 2. Adjusted Hazard Ratios for the Primary Outcome According to Six Groups Categorized by the Combination of Tonsillectomy and Type of Corticosteroid Therapy [file jamanetwopen-2-e194772-s001.pdf]

Supplementary Online Content

Hirano K, Matsuzaki K, Yasuda T, et al. Association between tonsillectomy and outcomes in patients with immunoglobulin A nephropathy. *JAMA Netw Open*. 2019;2(5):e194772. doi:10.1001/jamanetworkopen.2019.4772

eTable 1. Participating Facilities

eTable 2. Variables Used for Propensity Score Analysis

eTable 3. Baseline Characteristics of the Study Cohort Before and After Imputation

eTable 4. Comparison of Baseline Characteristics Between T0 and T1 Groups After IPTW

eTable 5. A Simple Multivariate Model for the Primary Outcome in Entire Cohort

eTable 6. Baseline Characteristics and Follow-up Data in Six Groups Categorized According to Tonsillectomy and Corticosteroid Therapy

eFigure 1. Rate of Patients Defined by Tonsillectomy and Corticosteroid Therapy According to Renin Angiotensin System Inhibitors

eFigure 2. Adjusted Hazard Ratios for the Primary Outcome According to Six Groups Categorized by the Combination of Tonsillectomy and Type of Corticosteroid Therapy

This supplementary material has been provided by the authors to give readers additional information about their work.

© 2019 Hirano K et al. *JAMA Network Open.*

eTable 1. Participating facilities

Hospital	Location
Asahikawa Medical University Hospital	Hokkaido
Fujita Health University Hospital	Aichi
Fukuoka University Hospital	Fukuoka
Gunma University Hospital	Gunma
Hamamatsu University School of Medicine, University Hospital	Shizuoka
Hokkaido University Hospital	Hokkaido
Iwate Prefectural Central Hospital	Iwate
Japanese Red Cross Nagoya Daini Hospital	Aichi
Juntendo University Hospital	Tokyo
Kanazawa Medical University Hospital	Kanazawa
Kanazawa University Hospital	Kanazawa
Kawasaki Medical School Hospital	Okayama
Kobe University Hospital	Hyogo
Kochi Medical School Hospital	Kochi
Kurume University Hospital	Fukuoka
Kyusyu University Hospital	Fukuoka
Nagasaki University Hospital	Nagasaki
Nagoya University hospital	Aichi
National Defense Medical College Hospital	Saitama
National Hospital Organization Hokkaido Medical Center	Hokkaido

© 2019 Hirano K et al. *JAMA Network Open.*

National Hospital Organization Kanazawa Medical Center	Kanazawa
Niigata University Medical & Dental Hospital	Niigata
Public Central Hospital of Matto Ishikawa	Ishikawa
Shiga University of Medical Science Hospital	Shiga
Shizuoka General Hospital	Shizuoka
Shonan Kamakura General Hospital	Kanagawa
Showa University Hospital	Tokyo
St· Luke’s International Hospital	Tokyo
St· Marianna University Hospital	Kanagawa
Tazuke Kofukai Foundation, Kitano Hospital	Osaka
Teikyo University Hospital	Tokyo
Teine Keijinkai Hospital	Hokkaido
The Jikei University Hospital	Tokyo
The University of Tokyo Hospital	Tokyo
Tohoku University Hospital	Miyagi
Tokyo Women’s Medical University Hospital	Tokyo
Toyama Prefectural Central Hospital	Tokyo
Toyonaka Municipal Hospital	Osaka
University of Fukui Hospital	Fukui
University of Miyazaki Hospital	Miyazaki
Wakayama Medical University Hospital	Wakayama
Yamagata University Hospital	Yamagata

© 2019 Hirano K et al. *JAMA Network Open.*

eTable 2. Variables used for propensity score analysis

Categories	Factors
Facility	Area of facility, category of hospital (university hospital or leading community hospital)
Basic case profile	Age, gender, year of renal biopsy, smoking, planning to bear children
Past history	Heart disease, cerebrovascular disease, peptic ulcer, osteoporosis, psychological disorder, hepatic disease, malignancy, tuberculosis
Comorbidity	Hypertension, diabetes mellitus
Family history	Kidney disease
Medications	Antihypertensive, lipid-lowering agent, hypoglycemic agent, Antihyperuricemic agent, antiplatelet agent, anticoagulant agent
Physical finding	Body mass index, mean arterial pressure
Laboratory test	eGFR, proteinuria, urine occult, uric acid, total cholesterol, IgA, complement C3
Corticosteroid therapy	Oral corticosteroid therapy, corticosteroid pulse therapy

© 2019 Hirano K et al. *JAMA Network Open.*

eTable 3. Baseline characteristics of the study cohort before and after imputation

Missing cases, number (%)	Baseline characteristics in finally enrolled 1,065 patients		
	Characteristics	Before imputation	After imputation
0 (0.0)	Age, median (IQR), years old	35 (25–52)	35 (25–52)
0 (0.0)	Women, No (%)	530 (49.8)	530 (49.8)
0 (0.0)	Diabetes mellitus, No (%)	49 (4.6)	49 (4.6)
11 (1.0)	BMI, mean (SD)	22.5 (3.5)	22.5 (3.5)
18 (1.7)	MAP, mean (SD), mmHg	92.3 (13.3)	92.3 (13.2)
0 (0.0)	eGFR, mean (SD), ml/min/1·73 m^2	76.6 (28.9)	76.6 (28.9)
31 (2.9)	Proteinuria, median (IQR), g/day	0.68 (0.28–1.31)	0.68 (0.29–1.30)
0 (0.0)	Urine occult 3+, No (%)	577 (54.2)	577 (54.2)
80 (7.5)	Uric acid, mean (SD) mg/dl	5.9 (1.6)	5.9 (1.5)
63 (5.9)	Total cholesterol, mean (SD), mg/dl	201.6 (48.1)	201.6 (46.9)
83 (7.8)	IgA, mean (SD), mg/dl	342.5 (129.9)	342.8 (125.4)
143 (13.4)	C3, mean (SD), mg/dl	101.8 (21.9)	102.0 (20.7)
0 (0.0)	RASi, No (%)	595 (55.9)	595 (55.9)

© 2019 Hirano K et al. *JAMA Network Open.*

0 (0.0)	Corticosteroid therapy, S0/S1/S2	574/204/287 (53.9/19.2/26.9)	574/204/287 (53.9/19.2/26.9)
0 (0.0)	Tonsillectomy, T0/T1	813/252 (76.3/23.7)	813/252 (76.3/23.7)

Abbreviations: BMI, body mass index; MAP, mean arterial blood pressure; eGFR, estimated glomerular filtration rate; RASi, renin-angiotensin aldosterone system inhibitors; S0, no steroid therapy; S1, oral steroid therapy without pulse regimen; S2, oral steroid therapy with pulse regimen; T0, patients who did not undergo tonsillectomy; T1, patients who underwent tonsillectomy

© 2019 Hirano K et al. *JAMA Network Open.*

eTable 4. Comparison of baseline characteristics between T0 and T1 groups after IPTW*

**Baseline characteristics**	**T1**	**T0**	***P* value**	**Std. Diff. (%)**
	**N = 252**	**N = 226**		
Age, median (IQR), years	29 (23–41)	28 (22–43)	.459	0.2
Women, N0(%)	146 (57.9)	122 (53.7)	.355	8.5
Diabetes mellitus, No (%)	6 (2.4)	5 (2.2)	.873	1.3
BMI, mean (SD)	22.1 (3.6)	22.0 (3.5)	.697	3.6
MAP, mean (SD), mmHg	89.2 (13.1)	88.5 (12.0)	.527	5.8
eGFR, mean (SD), ml/min/1·73 m^2	83.7 (29.1)	81.3 (29.8)	.368	8.2
Proteinuria, median (IQR), g/day	0.71 (0.33–1.30)	0.70 (0.34–1.43)	.726	8.5
Urine occult 3+, No (%)	153 (60.7)	153 (67.6)	.120	14.4
Uric acid, mean (SD), mg/dl	5.7 (1.6)	5.8 (1.4)	.579	5.1
RASi, No (%)	106 (42.1)	112 (49.6)	.097	15.1
Corticosteroid therapy, S0/S1/S2, No (%)	54/32/166	55/32/140	.641	7.6
	(21.4/12.7/65.9)	(24.2/14.0/61.7)		

Abbreviations: T1, patients who underwent tonsillectomy; T0, patients who did not undergo tonsillectomy; Std. Diff., standardized difference; BMI, body mass index; MAP, mean arterial blood pressure; eGFR, estimated glomerular filtration rate; RASi, renin-angiotensin aldosterone system inhibitors; S0, no steroid therapy; S1, oral steroid therapy without pulse regimen; S2, oral steroid therapy with pulse regimen

*ATT mode

© 2019 Hirano K et al. *JAMA Network Open.*

eTable 5. A simple multivariate model for the primary outcome in entire cohort

**Factors**	**HR (95% CI)**	**P value**
Age, per 10 years old	1.11 (0.97–1.28)	.130
Women versus men	1.16 (0.75–1.78)	.502
Diabetes mellitus *	1.57 (0.71–3.09)	.224
BMI, per 5 indexes	0.70 (0.50–0.98)	.038
MAP, per 10 mmHg	1.12 (0.95–1.33)	.168
eGFR, per 10 ml/min/1.73 m^2	0.83 (0.75–0.93)	.001
Proteinuria, per 1.0 g/day	1.25 (1.14–1.37)	<.001
Urine occult 3+ *	1.16 (0.79–1.70)	.448
Uric acid, per 1.0 mg/dl	1.22 (1.04–1.44)	.013
IgA, per 100 mg/dl	1.07 (0.92–1.24)	.398
C3, per 10 mg/dl	0.93 (0.85–1.02)	.135
RASi *	1.17 (0.76–1.84)	.483
S1 versus S0	0.35 (0.18–0.61)	.003
S2 versus S0	0.78 (0.45–1.30)	
T1 versus T0	0.43 (0.20–0.85)	.021

Abbreviations; HR, hazard ratio; CI, confidence interval; BMI, body mass index; MAP, mean arterial pressure; eGFR, estimated glomerular filtration rate; C3, complement 3; RASi, renin-angiotensin aldosterone system inhibitors; S0, no steroid therapy; S1, oral steroid therapy without pulse regimen; S2, oral steroid therapy with pulse regimen; T1, patients who iunderwent tonsillectomy; T0, patients who did not undergo tonsillectomy. *Yes versus No.

© 2019 Hirano K et al. *JAMA Network Open.*

eTable 6. Baseline characteristics and follow-up data in six groups categorized according to tonsillectomy and corticosteroid therapy

		T0			T1		
		**S0**	**S1**	**S2**	**S0**	**S1**	**S2**
		N = 520	N = 172	N = 121	N = 54	N = 32	N = 166
Baseline characteristics							
Age, median (IQR), years		41 (27–56)	35 (26–49)	35 (27–51)	30 (22–44)	26 (22–40)	30 (24–41)
Women, No (%)		233 (44.8)	93 (54.0)	58 (47.9)	35 (64.8)	13 (40.6)	98 (59.0)
Diabetes mellitus, No (%)		35 (6.7)	5 (2.9)	3 (2.5)	0 (0.0)	2 (6.3)	4 (2.4)
BMI, mean (SD)		22.9 (3.5)	22.2 (3.2)	22.4 (3.5)	22.1 (3.0)	21.8 (2.9)	22.2 (3.9)
MAP, mean (SD), mmHg		93.6 (13.2)	92.8 (13.1)	92.1 (12.7)	88.7 (14.8)	85.2 (8.5)	90.2 (13.2)
eGFR, mean (SD), ml/min/1·73 m^2		75.5 (28.8)	77.5 (26.6)	65.5 (28.7)	85.8 (27.0)	86.7 (29.2)	82.5 (29.8)
Proteinuria, median (IQR), g/day		0.54 (0.22–1.00)	0.86 (0.38–1.70)	1.36 (0.74–2.80)	0.30 (0.11–0.76)	0.90 (0.70–1.38)	0.76 (0.40–1.50)
Urine occult 3+, No (%)		236 (45.4)	108 (62.8)	80 (66.1)	26 (48.2)	22 (68.8)	105 (63.3)

© 2019 Hirano K et al. *JAMA Network Open.*

Uric acid, mean (SD), mg/dl		6.0 (1.6)	5.7 (1.5)	5.9 (1.4)	5.5 (1.9)	5.9 (1.6)	5.8 (1.5)
RASi, No (%)		313 (60.2)	93 (54.1)	83 (68.6)	21 (38.9)	13 (40.6)	72 (43.4)
Follow-up							
Period, median (IQR), years		5.7 (1.9–8.5)	7.6 (2.8–9.1)	5.2 (1.8–8.5)	4.3 (1.1–7.9)	7.7 (4.2–8.3)	5.1 (1.7–8.2)
Primary outcomes, No (%)		82 (15.8)	12 (7.0)	24 (19.8)	2 (3.7)	1 (3.1)	8 (4.8)
Additional therapy, No (%) RASi/S/RASi+S		33/46/12	19/7/3	15/0/0	3/2/0	2/1/0	10/0/0
		(6.4/8.9/2.3)	(11.1/4.1/1.74)	(12.4/0.0/0.0)	(5.6/3.7/0.0)	(6.3/3.1/0.0)	(6.0/0.0/0.0)
Death, No (%)		3 (0.6)	2 (1.2)	1 (0.8)	0 (0.0)	0 (0.0)	0 (0.0)

Abbreviations: T0, patients who did not undergo tonsillectomy; T1, patients who underwent tonsillectomy; S0, no corticosteroid therapy; S1, oral corticosteroid therapy without pulse regimen; S2, oral corticosteroid therapy with pulse regimen; BMI, body mass index; MAP, mean arterial blood pressure; eGFR, estimated glomerular filtration rate; RASi, renin-angiotensin aldosterone system inhibitors; S, corticosteroid therapy

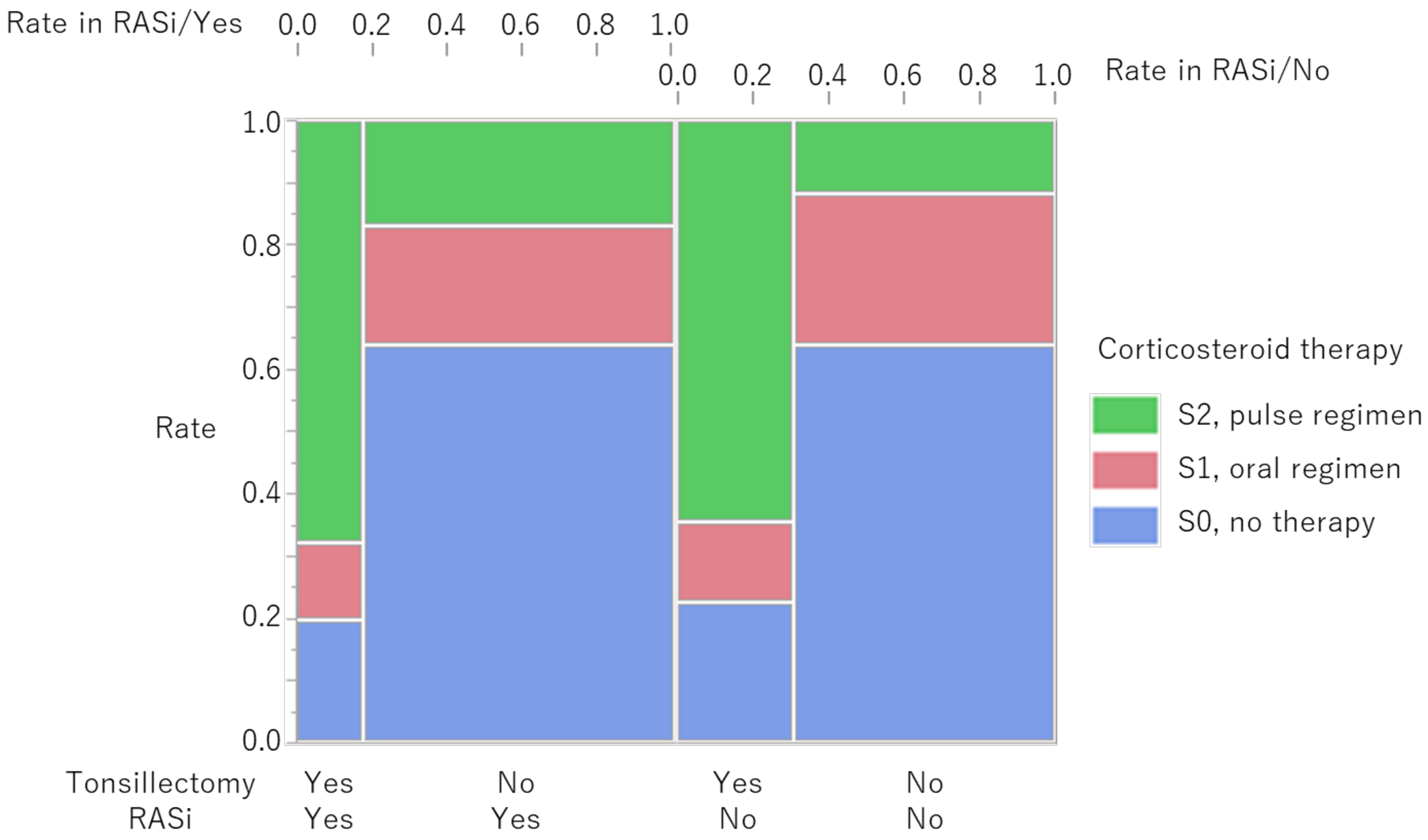

eFigure1. Rate of patients defined by tonsillectomy and corticosteroid therapy according to renin angiotensin system inhibitors

© 2019 Hirano K et al. *JAMA Network Open.*

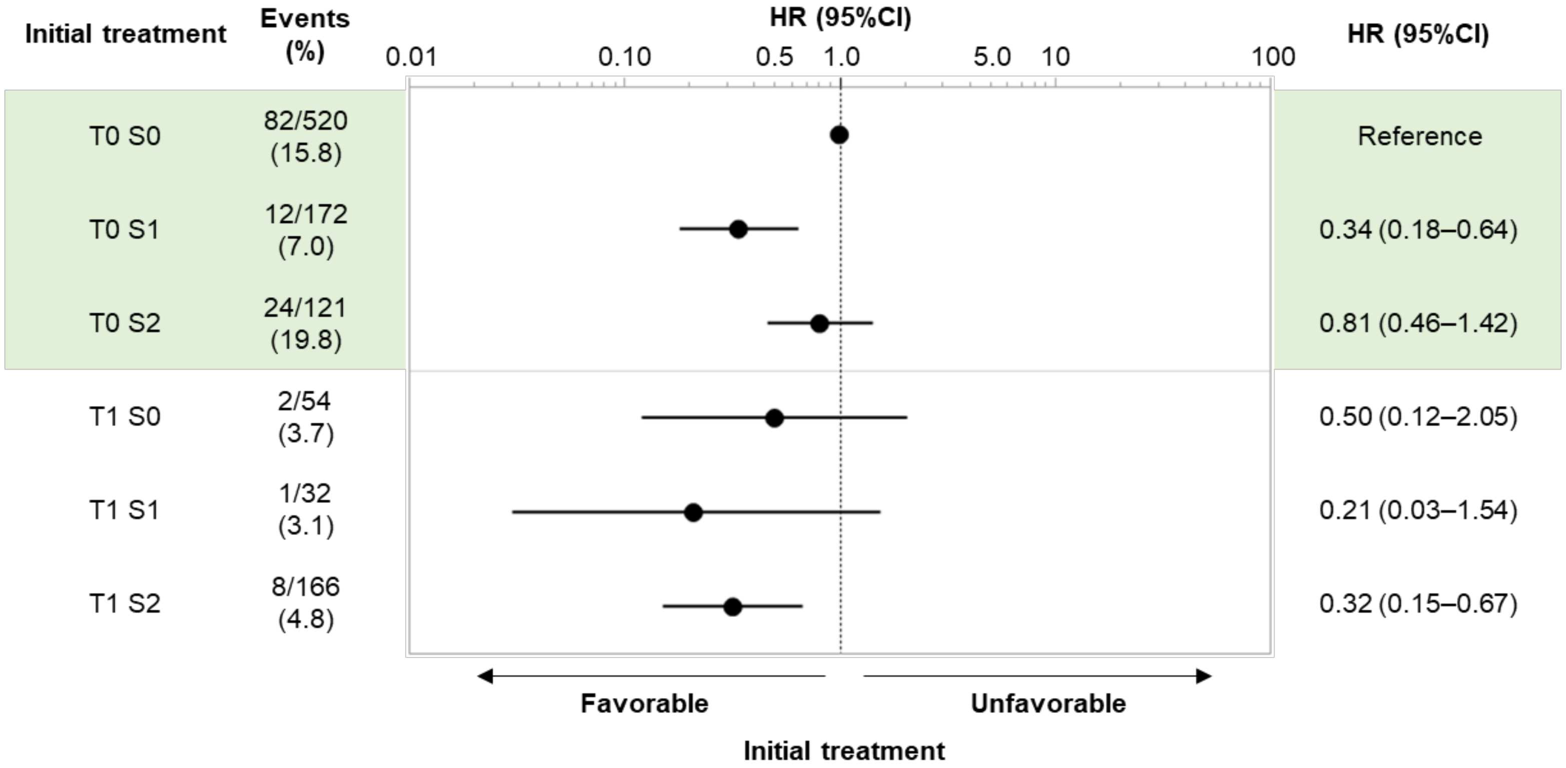

eFigure 2. Adjusted hazard ratios for the primary outcome according to six groups categorized by the combination of tonsillectomy and type of corticosteroid therapy.

The graphic shows HRs for the primary outcome in the six initial treatment categories; T0S1, T0S2, T1S0, T1S1, T1S2, and T0S0 (reference). The HRs were adjusted according to age, gender, body mass index, diabetes mellitus presence/absence, mean arterial pressure, estimated glomerular filtration rate, proteinuria, urine occult, uric acid, total cholesterol, immunoglobulin A, complement 3, and renin-angiotensin system inhibitor use. Abbreviations; T0, patients who did not undergo tonsillectomy; T1, patients who underwent tonsillectomy; S0, no corticosteroid therapy; S1, oral corticosteroid therapy without pulse regimen; S2, oral corticosteroid therapy with pulse regimen; HR, hazard ratio.

© 2019 Hirano K et al. *JAMA Network Open.*
